# Ergonomics in Knee Arthroscopy: A Scoping Review

**DOI:** 10.1002/ars2.70111

**Published:** 2026-10-01

**Authors:** Mariam Alemi, Yousof Alemi, Martinique Vella‐Baldacchino

**Affiliations:** ^1^ Faculty of Medicine Imperial College London London UK; ^2^ North West Anglia NHS Foundation Trust Peterborough UK; ^3^ MSk Lab, Department of Surgery & Cancer Imperial College London London UK

## Abstract

**Purpose:**

To evaluate the existing evidence regarding ergonomics in knee arthroscopy, including surgeon positioning, limb manipulation strategies, support devices, and reported ergonomic burden.

**Methods:**

This scoping review was conducted in accordance with the Arksey and O’Malley methodological framework and the Preferred Reporting Items for Systematic Reviews and Meta‐Analyses extension for Scoping Reviews guidelines. A literature search of PubMed, Embase, and Scopus was performed from database inception to December 2025. Eligible studies addressed ergonomic considerations in knee arthroscopy. Two independent reviewers screened titles, abstracts, and full texts, and findings were synthesized narratively.

**Results:**

The search identified 1013 records, of which 8 met the inclusion criteria. The literature consisted predominantly of descriptive technical reports, alongside cross‐sectional questionnaires and 1 observational study. Routine knee arthroscopy involved sustained valgus and varus stress applied to the limb and non‐neutral postures maintained by surgeons or assistants. Positioning aids and technical innovations have been proposed; however, none have undergone objective ergonomic assessment. Surgeon‐reported data suggested that knee arthroscopy is ergonomically challenging and frequently associated with musculoskeletal discomfort.

**Conclusions:**

The review identified 8 studies examining ergonomics in knee arthroscopy, which predominantly consisted of descriptive technical reports lacking objective ergonomic assessment. Surgeon‐reported data indicated that knee arthroscopy is frequently perceived as ergonomically challenging, with musculoskeletal discomfort most commonly affecting the lower back and shoulders.

**Clinical Relevance:**

Knee arthroscopy places substantial physical demand on surgeons. Mapping the ergonomic challenges and limitations associated with arthroscopy may help inform future device innovation aimed at reducing work‐related musculoskeletal injury.

## Graphical abstract

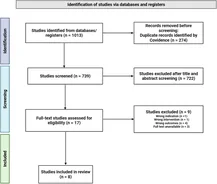

Knee arthroscopy is a widely used procedure in orthopaedic surgery that plays an integral role in the operative management of intra‐articular knee pathologies. Arthroscopy offers both diagnostic and therapeutic roles in the knee for conditions including meniscal tears, ligament injuries, and chondral lesions.^1^ It is regarded as the standard of care in meniscal injury, which is among the commonest knee injuries,^2^ owing to its high diagnostic accuracy and capacity to facilitate minimally invasive yet targeted interventions.

Unlike other joints used in arthroscopy, the knee joint is compact and ligamentously constrained.^3^ Consequently, direct visualization of knee compartments requires deliberate manipulation of the joint, often involving knee flexion, tibial rotation, and the application of valgus or varus stress.^3^ Although traction devices can be used in the hip^4^ or shoulder^5^ to apply longitudinal traction and distract the joints, thereby increasing joint space, the knee relies on mechanically applied forces to allow for proper exposure. Valgus stress tensions the medial collateral ligament exposing the medial meniscus, and varus stress exposes the lateral meniscus by tensioning the lateral collateral ligament.^3^ These configurations can be combined with the “figure‐of‐4” position, whereby the hip is externally rotated and the ankle is rested over the contralateral knee, further opening the lateral joint compartment.^3^

Although arthroscopic procedures each present their own technical demands, knee arthroscopy is distinguished by its reliance on surgeon‐applied forces to achieve joint exposure, rather than passive distraction mechanisms available in hip and shoulder arthroscopy. Commonly, positioning aids have been incorporated into arthroscopy to support limb stabilization: circumferential thigh holders, lateral thigh posts,^6^ or support from stools.^7^ Despite providing some assistance, these offer limited continuous stability and still rely on the surgeon to maintain limb position and apply valgus, varus, or rotational forces while operating the arthroscope. Alternatively, an assistant can exert these forces, although this is frequently impractical for prolonged periods.^1^ More recently, emerging descriptions of a wearable arthroscopic belt have been proposed to support positioning, although early descriptions suggest ongoing requirements for dynamic adjustment and limb manipulation.^8^ As such, knee arthroscopy remains largely dependent on surgeon‐applied manipulation, perpetuating the ergonomic challenge of achieving comprehensive visualization across knee compartments.

Work‐related musculoskeletal (MSK) pain and injury are highly prevalent among orthopaedic surgeons, attributed largely to strenuous postures and physical loads associated with limb manipulation.^9^, ^10^, ^11^ Although arthroscopy remains comparatively understudied ergonomically, evidence from laparoscopy shows an increased MSK symptom burden relative to open surgery.^12^ Given analogous risk factors of minimally invasive surgery—sustained non‐neutral postures and static muscle loading—arthroscopic surgeons are similarly predisposed to MSK strain.^13^ This concern is reflected in orthopaedics: a 2022 survey reported that 86% of orthopaedic surgeons experienced at least 1 MSK condition, with an average of 2 conditions per surgeon which were frequently attributed to positioning and instrument use.^14^ A further cross‐sectional study reported that approximately 20% of orthopaedic surgeons required surgery for work‐related MSK injuries and also highlighted associated occupational and psychological sequelae, including 64.4% experiencing features of burnout.^15^ Despite the prevalence of MSK burden and long‐term complications, the ergonomic implication of knee arthroscopy remains poorly defined. The purpose of this scoping review was to evaluate the existing evidence regarding ergonomics in knee arthroscopy, including surgeon positioning, limb manipulation strategies, support devices, and reported ergonomic burden. We hypothesized that the existing literature would be sparse and methodologically weak, with few studies employing objective ergonomic assessment and minimal innovation in support devices.

## METHODS

### Study Design

This scoping review followed the Arksey and O’Malley framework,^16^ further developed by Levac et al.^17^ Reporting followed the Preferred Reporting Items for Systematic Reviews and Meta‐Analyses extension for Scoping Reviews guidelines.^18^ The review protocol was registered on the Open Science Framework, and no methodological changes were made (https://osf.io/javsb).

### Search Strategy

A comprehensive search of PubMed, Embase, and Scopus was carried out in December 2025, restricted to English‐language and full‐text articles (Supporting Information). The search strategy incorporated keywords and vocabulary relating to ergonomics, MSK strain, positioning/support devices, knee arthroscopy, and surgeon fatigue. The full Boolean string is provided in the Supporting Information, with adaptations made for Embase and Scopus. Citations were imported into Covidence^19^ for deduplication and structured screening.

### Eligibility Criteria

Eligible studies addressed ergonomic considerations relevant to knee arthroscopy, encompassing descriptions of surgeon positioning, limb manipulation strategies, use of support devices, or reported MSK symptoms and fatigue associated with the procedure. Studies were excluded if they focused on nonarthroscopic procedures, examined ergonomics in surgical specialties unrelated to orthopaedic arthroscopy, or were review articles, conference abstracts, or non‐English publications. Exclusion criteria also included studies without full‐text availability. Title and abstract screening were followed by full‐text review, performed independently by 2 reviewers, with conflicts resolved through discussion and consensus.

### Quality Assessment

Formal quality appraisal was not performed, as per scoping review methodology. Nonetheless, methodological quality was considered narratively during synthesis, noting the predominance of descriptive technical reports and the absence of validated ergonomic outcome measures or comparative designs. No studies reported outcomes disaggregated by sex, precluding analysis in line with Sex and Gender Equity in Research guidelines.

## RESULTS

The literature search identified 1013 records, of which 274 were removed as duplicates, leaving 739 articles screened by title and abstract. Seventeen full‐text studies were assessed for eligibility, resulting in 8 studies being included in the final synthesis (Figure 1).

**FIGURE 1:**
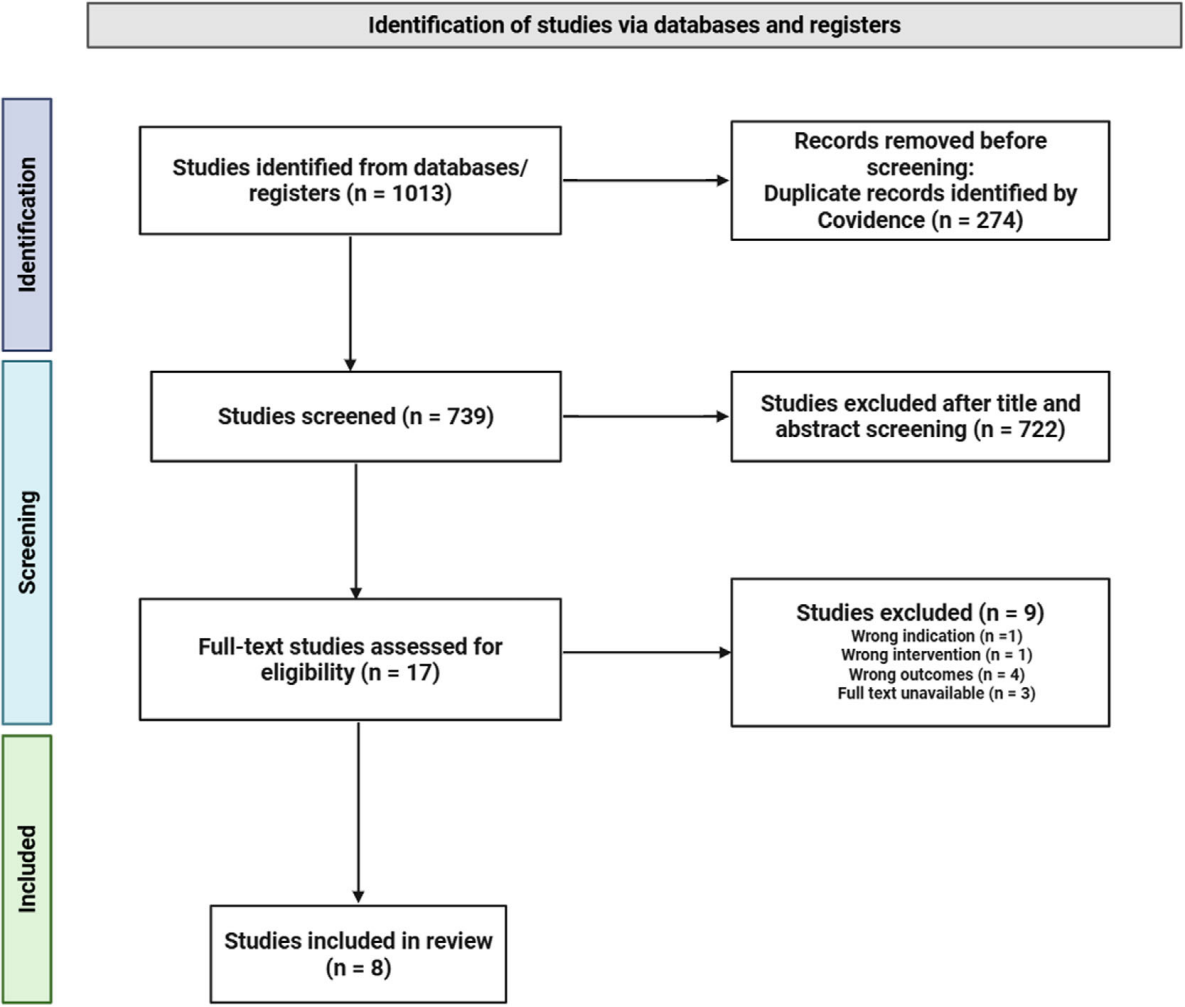
PRISMA flow diagram illustrating the study selection process. Of the 1013 records identified across PubMed, Embase, and Scopus, 8 studies met the inclusion criteria after deduplication, title and abstract screening, and full‐text review. (PRISMA, Preferred Reporting Items for Systematic Reviews and Meta‐Analyses.)

The included studies were published between 1986 and 2025 and reflect a predominantly descriptive evidence base. Study designs consisted of technical reports describing surgeon positioning during knee arthroscopy or the use of support devices (n = 6), a cross‐sectional surgeon questionnaire (n = 1), and an observational study with qualitative interviews (n = 1). None of the studies incorporated quantitative analysis of ergonomic or biomechanical outcome measures, or comparative study designs.

The primary focus of the studies was the description of positioning techniques or technical modifications used during knee arthroscopy. Ergonomic considerations were addressed implicitly or qualitatively rather than as predefined study outcomes; findings were therefore synthesized narratively. The characteristics and thematic classification of studies are summarized in Table 1.

**TABLE 1:** Summary of Included Studies Assessing Ergonomics in Knee Arthroscopy

**Author**	**Study Design**	**Thematic Category**	**Primary Focus**	**Ergonomic Considerations**	**Key Findings**	**Limitations**	**Level of Evidence**
Dunbar^20^	Technical note	Optimizing ergonomic position	Improvised nonmechanical thigh holding method	Assistant fatigue and maintaining adequate joint exposure	Describes a “very simple” thigh holder using adhesive tape and 2 pieces of 8‐ft muslin lengths. Notes assistants would become “fatigued during long cases” from manipulating the leg and applying valgus/varus forces leading to the development of this solution	Simply describes innovation and no acknowledgement of musculoskeletal outcomes or posture in the surgeon	N/A—technical note
Ward and Lubowitz^3^	Technical positioning guide	Routine positioning and technique	Reviews routine patient positioning	Hip bracing; assistant vs surgeon‐applied valgus/varus forces	Describes using a circumferential leg holder or lateral post; assistant applies valgus or varus forces. When operating alone, the surgeon pushes the leg onto their hip to position into valgus/varus providing compartment exposure	Ergonomic burden implied without ergonomic metrics/outcomes. Strategies not compared	N/A—technical note
Erduran et al.^21^	Technical brief	Optimizing ergonomic position	Modifying ergonomic positioning	Musculoskeletal load on surgeons	Explicit ergonomic intent: describes replacing leg holder with pelvic support at the femoral level with a 2‐3 step ladder vs conventional approach, whereby surgeon presses patient leg between their own body and lower limb while applying pushing forces. Reduced need for assistance	Descriptive; no objective outcome assessment or comparative outcomes	N/A—technical note
Cooper^22^	Technical update	Routine positioning and technique	Single‐portal technique	Workflow ergonomics; dependence on assistants	Outlines single‐portal arthroscopic technique although a disadvantage is “the learning curve of different ergonomics.” Describes assistants as essential—“needed for proper knee positioning”	Ergonomic outcomes not measured; no posture/fatigue outcomes	N/A—technical note
Jaiprakash et al.^23^	Cross‐sectional survey	Surgeon‐reported ergonomic burden	Surgeon attitudes and limitations (n = 93)	Ergonomic difficulty self‐reported by surgeons and pain experienced after performing procedures	59.1% found knee arthroscopy ergonomically challenging; 22.6% reported physical pain after knee arthroscopy—of these, 50% were lower back pain and 30% shoulder pain; 73.1% reported frustration with arthroscopic equipment	Self‐report; lack of objective biomechanical measurements	Level IV
Opie et al.^24^	Observational study + interviews	Surgeon‐reported ergonomic burden	Workflow demands during knee arthroscopy	Strategies of physically maneuvering leg during surgery; ergonomic discomfort with qualitative evidence	Quantifies time spent by surgeons manipulating leg position: mean 3.43% of time involved in physical maneuvering while simultaneously handling instruments in both hands and visualizing knee through a monitor. Taxing positions and high cognitive effort required, where you can get into “quite awkward positions, sometimes for a while”	Small sample size (knee surgery n = 15, surgeon interviews n = 2); not an ergonomic biomechanics study	Level IV
Ren et al.^7^	Technical note	Optimizing ergonomic position	Optimizing knee positioning	Surgeon comfort and effort; use of assistants	Describes valgus force maintained by assistant or supported by post + surgeon hip. Operating solo means surgeon must “sacrifice comfort” to “exert additional expenditure of effort.” Proposes use of a metal round stool to assist with positioning for posteromedial access	Descriptive; no ergonomic outcomes assessed; no comparative assessment	N/A—technical note
Luttjeboer et al.^8^	Technical innovation	Novel ergonomic innovation	Wearable leg support (Knee Arthroscopy Belt)	Lower back pain, posture and reduced manual support from assistants	Explicit mention of lower back pain experienced by surgeons because of prolonged static postures. Describes a belt worn under sterile gown to support leg and “optimise limb positioning…eliminating the need for a surgical footrest and reducing the need for manual leg support”	Early‐stage innovation that is purely descriptive; no comparative trials or ergonomic outcomes measured	N/A—technical note

N/A, not applicable.

Routine positioning and limb manipulation practices were consistently described in the literature as key components of knee arthroscopy.^3^, ^7^, ^22^ Commonly reported techniques included the use of circumferential thigh holders or lateral thigh posts for limb stabilization, combined with manual manipulation of the knee into valgus or varus.^3^, ^7^ Maneuvers such as the figure‐of‐4 were frequently described to facilitate compartmental access.^3^

Limb manipulation was often performed by the surgeon or a surgical assistant where available.^3^, ^7^, ^24^ These practices were documented descriptively in the literature; however, none of the studies formally assessed ergonomic factors such as surgeon posture, physical workload, or MSK strain.

Support devices and positioning aids have been presented as adjuncts to routine practice, intended to support the limb and/or reduce surgeon/assistant fatigue associated with prolonged manual manipulation.^7^, ^8^, ^21^, ^22^ Proposed solutions included pelvic supports, foot ladders or stools,^7^, ^21^ and a wearable limb‐support belt.^8^ Evaluation of these devices was limited to descriptive accounts, again without incorporation of objective ergonomic outcome measures.

Two of the studies addressed surgeon perspectives on current practice in knee arthroscopy.^23^, ^24^ A cross‐sectional questionnaire reported that the procedure was commonly perceived as ergonomically challenging, with physical discomfort often reported in the lower back and shoulders.^23^ Surgeons expressed dissatisfaction with existing arthroscopic equipment and positioning strategies. A further study documented non‐neutral positions sustained during arthroscopy, using video analysis of 15 procedures.^24^ Collectively, these highlight surgeon‐perceived ergonomic burden during knee arthroscopy, although neither study employed objective assessment frameworks to quantify this.

## DISCUSSION

The most important finding of this study is that ergonomics in knee arthroscopy remains critically under‐researched, with this scoping review identifying only 8 relevant studies, of which 6 were technical reports lacking objective ergonomic evaluation. The findings of this review reveal a mismatch between the well‐recognized physical demands of the procedure and the paucity of research dedicated to the field. Knee arthroscopy occupies a unique position within orthopaedic surgery, combining minimally invasive access with substantial physical demands placed on the surgeon. Routine practice depends on sustained limb positioning and hip bracing, asymmetric postures, and the simultaneous execution of fine and gross motor tasks. These demands may be further amplified by patient‐related factors such as body habitus, whereby limb manipulation in patients with higher body mass requires increased and prolonged exertion.^25^, ^26^, ^27^ Despite this, the physical demands inherent to knee arthroscopy are implicitly embedded within standard practice, with ergonomic considerations seldom examined or incorporated into technical descriptions.

Although procedural demands can vary depending on assistant availability, surgeons are often required to independently maintain limb position and concurrently control arthroscopic instruments. This multifaceted task requirement represents an ergonomic challenge unique to knee arthroscopy that remains under‐addressed in the literature. This could be partly explained by the predominance of technical reports within this field, designed to show feasibility or reproducibility, with success defined by execution of the procedure, not surgeon‐related outcomes. As a result, physical strain associated with forceful limb manipulation is often normalized as an assumed requirement of the procedure, rather than recognized as a modifiable risk factor warranting systematic ergonomic evaluation.

The occupational impact of these ergonomic demands is reflected in surgeon‐reported data. Available data indicate that 59.1% of surgeons report the procedure to be ergonomically challenging, with discomfort commonly affecting the lower back and shoulders.^23^ This is consistent with the sustained non‐neutral postures and exertional limb manipulation required during the standard knee arthroscopy procedure.

Evidence from other minimally invasive surgical specialties, including endoscopy and laparoscopy, provides important contextualization for these observations. A substantial body of laparoscopic literature shows a strong association between work‐related MSK injuries and minimally invasive practice. Studies identify unnatural body postures and prolonged static muscle loading as notable ergonomic hazards.^28^, ^29^, ^30^, ^31^, ^32^, ^33^ Across surgical subspecialties, ergonomic strain has been linked to meaningful occupational consequences. Over 50% of surgeons report using analgesics for work‐related pain,^34^ and up to 33% of orthopaedic surgeons experiencing work‐related MSK disorders report leave of absence, practice restriction or modification, or early retirement.^35^

Although the procedural mechanics of other minimally invasive procedures differ from knee arthroscopy, the translational relevance of this evidence is justified on the basis of shared biomechanical risk factors rather than procedural similarity, including prolonged static postures and loading, asymmetric non‐neutral positions, and concurrent fine motor control under force. Given this, evidence from minimally invasive surgery may provide valuable contextualization for knee arthroscopy, which is relatively under‐researched. The nature of orthopaedic surgery is characterized by repetition and extremes of motion, making it a particularly physically demanding specialty.^36^, ^37^, ^38^ When this intrinsic risk is combined with the ergonomic challenges that come with minimally invasive techniques, arthroscopic orthopaedic surgeons may be particularly prone to cumulative occupational strain. Beyond individual morbidity, work‐related MSK pain has broader workforce and system‐level consequences. Absenteeism because of occupational injury has been reported in 12% of surgeons, with a substantial proportion requiring prolonged leave exceeding 1 month.^15^ NHS workforce data also show higher rates of MSK‐related absence among senior specialty doctors compared with junior doctors, likely a reflection of cumulative surgical exposure.^39^ Addressing modifiable ergonomic risk factors may therefore have system‐level implications, contributing not only to preservation of surgical capacity but also to the long‐term sustainability of health care delivery.

The evidence gaps identified in this review highlight clear priorities for future investigation. Future research should prioritize formal ergonomic evaluation of both routine practice and emerging technologies using objective assessment frameworks. In other minimally invasive surgical specialities, ergonomic risk has been quantified using tools such as Rapid Upper Limb Assessment,^40^ Rapid Entire Body Assessment,^41^ surface electromyography,^29^ and wearable sensor technologies,^42^ enabling direct comparison of pre‐ and postinterventional ergonomic profiles.^43^, ^44^, ^45^, ^46^ Such approaches may also help characterize the psychosocial burden associated with work‐related MSK injury,^47^, ^48^ supporting the development of ergonomic guidelines promoting occupational sustainability and improving surgeon working conditions.^49^

### Limitations

Several limitations of this review should be acknowledged. The literature available was sparse and predominantly descriptive with ergonomic outcomes often addressed implicitly in qualitative studies rather than examined as predefined outcomes in interventional or comparative study designs. No studies employed validated ergonomic assessment strategies or biomechanical measures, and there was a lack of comparative evaluations of positions or support devices.

The review was also limited to English‐language publications. Background evidence contextualizing the occupational burden of orthopaedic surgery draws largely on studies from the United States, and reported rates of MSK injury may be variable across international settings. The predefined scope of this review limited the yield of the search strategy, reflecting the early developmental stage of research in this field rather than deficiencies in review design. Furthermore, none of the included studies reported outcomes stratified by sex, limiting analysis in accordance with Sex and Gender Equity in Research guidelines.

## CONCLUSIONS

The review identified 8 studies examining ergonomics in knee arthroscopy, which predominantly consisted of descriptive technical reports lacking objective ergonomic assessment. Surgeon‐reported data indicated that knee arthroscopy is frequently perceived as ergonomically challenging, with MSK discomfort most commonly affecting the lower back and shoulders.

## SUPPORTING INFORMATION

Additional supporting information can be found online in the Supporting Information section.

## DISCLOSURES

The authors (M.A., Y.A., M.V‐B.) declare that they have no known competing financial interests or personal relationships that could have appeared to influence the work reported in this paper.

## Supporting information

Supplementary Material
