# Supplementary material for: Ergonomics in Knee Arthroscopy: A Scoping Review
Source: Arthrosc Sports Med Rehabil. 2026 Oct 1;8(5):e70111. doi: 10.1002/ars2.70111 (PMC13629507; doi:10.1002/ars2.70111)
Supplement: Supplementary file 1 [file ARS2-8-e70111-s001.pdf]

Appendix

PubMed Search Strategy

["knee arthroscopy"[Title/Abstract] OR "arthroscopic knee surgery"[Title/Abstract]]

AND

[ergonomic*[Title/Abstract] OR posture[Title/Abstract] OR musculoskeletal[Title/Abstract] OR fatigue[Title/Abstract] OR discomfort[Title/Abstract] OR "work-related injury"[Title/Abstract] OR "surgeon strain"[Title/Abstract] OR "surgeon fatigue"[Title/Abstract] OR positioning[Title/Abstract] OR "leg holder"[Title/Abstract] OR "support device"[Title/Abstract]]

This search strategy was adapted for Embase and Scopus to account for specific database syntax and indexing.
